# Agar and Chitosan Hydrogels’ Design for Metal-Uptaking Treatments

**DOI:** 10.3390/gels10010055

**Published:** 2024-01-11

**Authors:** Luana Cuvillier, Arianna Passaretti, Elodie Guilminot, Edith Joseph

**Affiliations:** 1 Laboratory of Technologies for Heritage Materials, University of Neuchâtel, Bellevaux 51, 2000 Neuchâtel, Switzerland; luana.cuvillier@he-arc.ch (L.C.); arianna.passaretti@he-arc.ch (A.P.); 2 Haute Ecole Arc Conservation Restauration, University of Applied Sciences and Arts Western Switzerland HES-SO, Espace de l’Europe 11, 2000 Neuchâtel, Switzerland; 3 Arc’Antique Conservation and Research Laboratory, 26 Rue de la Haute Forêt, 44300 Nantes, France; elodie.guilminot@loire-atlantique.fr

**Keywords:** agar, chitosan, cryo–SEM, spectroscopy, rheology, deferoxamine, silver

## Abstract

In the field of cultural heritage, the use of natural gels is rising for the application of active agents. Here, two natural polymers are assessed: agar, a pioneer hydrogel for conservation treatments, and chitosan, a rather novel and metal-binding gel. For chitosan, a state-of-the-art based formulation (CS–ItA–LCys) is evaluated as it was reported for silver-complexing properties. It is evaluated whether these polymers can withstand the addition of the chelating compound deferoxamine, which is a bacterial siderophore. This allows for the obtainment of completely bio-sourced gel systems. A Fourier-transformed (FT) infrared spectroscopy characterization is performed, completed with rheological measurements and Cryo-Scanning Electron Microscopy (cryo–SEM) to investigate the physico–chemical properties of the gels, as well as their interaction with deferoxamine. Both polymers are also tested for their inherent complexing ability on silver ions using FT–Raman spectroscopy. A multi-analytical comparison shows different microstructures, in particular, the presence of a thick membrane for chitosan and different mechanical behaviors, with agar being more brittle. Neither hydrogel seems affected by the addition of deferoxamine; this is shown by similar rheological behavior and molecular structures in the presence or absence of the chelator. The intrinsic abilities of the chitosan formulation to make silver complex are demonstrated with the observation of two peaks characteristic of Ag–S and Ag–O bonds. Agar and chitosan are both proven to be reliable gels to act as carriers for bio-based active agents. This paper confirms the potential asset of the chitosan formulation CS–ItA–LCys as a promising gel for the complexation of soluble silver.

## 1. Introduction

Among the different treatments commonly used in heritage conservation, metal uptake is widely studied over a large range of materials, and for several purposes. Indeed, it is used to remove iron or copper stains (e.g., marble, paper, textile) [1,2,3], remediate waterlogged archeological wood salt contaminations [4], and remove corrosion on metal pieces to improve the aesthetics or readability of the objects [5,6,7]. In parallel, hydrogels are becoming a must-have in heritage conservators’ workshops as a delivery system for interventions. Their interesting properties allow a precise and selective treatment, including on vertical or other complex surfaces [8]. Gel materials allow a more precise application, which means a lower amount of treating solution or water is required. This can be problematic for water-sensitive artifacts such as composite objects, for instance [9]. Amongst the different types of gels studied, it is trendy to use materials extracted from natural resources, in particular, polysaccharides (e.g., gellan or agar) [9].

To ascertain their innocuousness and possible application for the preservation of heritage, gels deserve and need more studies. In particular, the definition of accurate preparation protocols and the evaluation of compatibility between the gel components (i.e., polymers and chelators for metal uptake) should be achieved.

Agar is one of the most-used delivery systems in cultural heritage [10,11,12,13,14,15]. It is bioderived as it is extracted from red seaweed membranes [16]. It is appreciated by conservators thanks to good mechanical and retention properties [11,17]. Moreover, it is easily accessible, affordable, and peelable, implying an ease of removal. Some publications mention its complexing abilities without demonstrating it for iron [18] or copper [19].

Another compound, chitosan, has received attention recently. It is naturally derived from crustaceans or fungi and has been praised in past decades, especially in the medical field or for heavy metal remediation [20,21]. Recently, applications in cultural heritage have been found for chitosan due to its protective assets, which include operating as coating for copper and silver [22,23,24] and consolidating anti-microbial agents on various substrates (i.e., paper, paintings, textiles, metals, wood) [25,26,27,28,29,30]. Chitosan has been used in the form of gel film or as nanoparticles or nanocomposites with ZnO [26,31]. To the best of our knowledge, the use of chitosan as a cleaning gel in the cultural heritage conservation field has been reported only once, for the removal of manganese stains on glass [32].

Regarding its properties as a gelling agent for conservation treatment, it is to be noted that it can be prepared in a peelable way using alkaline solutions [21], thus reducing the chance of residues when removing the gel after treatment, which is of concern for heritage conservation professionals [33]. In addition, chitosan is a known compound for the complexation of heavy metals and, in particular, copper ions [34,35]. The abundant amino and hydroxyl groups are responsible for this ability [36,37]. More recently, it was also found that it has the ability to chelate iron [38]. As such, it is a promising gelling agent to study for metal uptake.

Interestingly, an easy-to-prepare chitosan-based formulation, including L–Cysteine and itaconic anhydride (CS–ItA–LCys), has been reported to have silver complexing abilities [39]. This is of great interest for cultural heritage conservation where the removal of silver remains a topic with no definite solution [40]. Nevertheless, the reported article demonstrates uptake abilities of the gel but not the occurrence of a complexing reaction with silver ions. Uptake could then be related to absorption properties of chitosan gel. The interactions between gel networks and metal ions in cultural heritage are of novel research interest. They have been addressed in few papers, mainly on copper [41], highlighting the need for further studies.

In parallel, the compatibility between the chosen active agents and the used hydrogel is a key question to be raised before the application of any three-pronged formulation (water–active agents–polymer) is studied. Indeed, it is sometimes not possible to combine them. For instance, the need for a heating step for agar preparation could prevent the use of some thermosensitive complexing compounds [42], or there could be a lack of suitable pH ranges between the compounds that would result in the modification of the physico–chemical properties of the selected gel.

This article first evaluates the two above-mentioned polymers, comparing the properties of agar and chitosan as pioneer and novel hydrogels for cultural heritage purposes. Their mechanical and structural properties are compared using a multi-analytical approach, including ATR–Fourier Transformed Infrared (ATR–FTIR), rheological measurements, swelling evaluation, and cryo-Scanning Electron microscopy (cryo–SEM).

In a second step, this article examines gel–chelator interactions, focusing on agar and chitosan with the addition of a selected chelating agent, a microbial siderophore called deferoxamine (DFO). This active agent was selected as its metal uptake properties have been praised in the cultural heritage field on several substrates (e.g., paper, wood, metal, textiles) [17,43,44], and allows for the achievement of bio-based formulations. In addition, this chelator shows high biodegradability in the environment, which means it has a reduced ecological impact compared to EDTA, a chelator commonly used in the heritage conservation field [45]. Nevertheless, it is worth noting that regarding health hazards, deferoxamine is used as a medical treatment for iron and aluminum overloads. Thus, some care should be used when handling this compound [46].

Finally, formulations are tested for their complexation ability with silver. In particular, the CS–ItA–LCys formulation described in the literature is more closely examined as we try to provide new insights on its molecular structure and possible interaction with silver.

## 2. Results and Discussion

### 2.1. Comparison of Classical Agar versus Novel Chitosan Gel Formulations

#### 2.1.1. Structural Properties of the Polymers’ Networks

**Figure 1:**
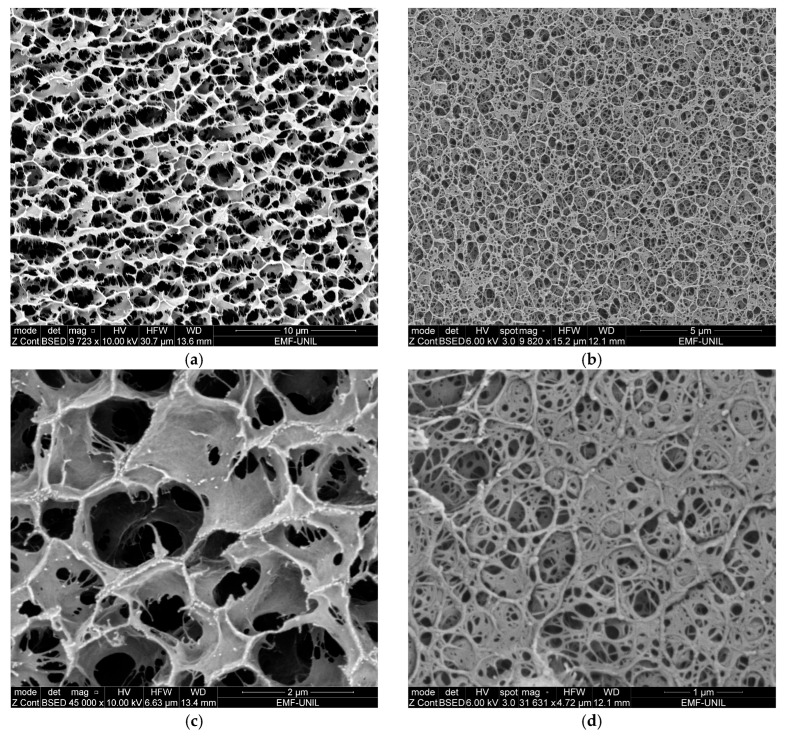
Cryo–SEM observations of (**a**) 3.3% (by mass) CS–ItA–LCys gel (**b**) 3% (by mass) agar gel and at higher magnification for (**c**) CS–ItA–LCys gel and (**d**) agar gel.

From the obtained cryo-SEM images, agar and chitosan polymers show different structural appearances. The agar microstructure is overall denser (Figure 1b), with pores that are quite regular and connections between the polymer chains resembling threads (Figure 1d). Agar gelification is known to be achieved by the transformation from a fluctuating disordered coil conformation in the solution to a rigid, ordered, co-axial structure that forms connections between the double helices in the gel network [47,48], resulting in precise junction points between threads, as observed on Figure 1b,d.

**Figure 2:**
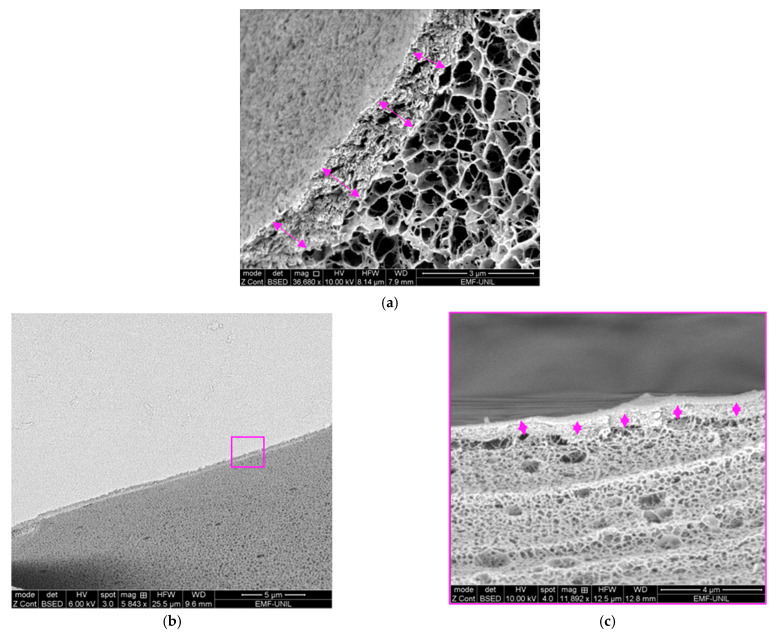
Cryo–SEM membrane observations of (**a**) 3.3% (by mass) plain CS–ItA–LCys gel (**b**) 3% (by mass) plain agar gel. Membrane thickness is indicated with purple double arrows. A close-up of the agar membrane is observed in the purple square (**c**).

The CS–ItA–LCys structure is less neat and homogeneous, with large pores that are less defined, hindering measurements of the average pore diameter (Figure 1a). The separation between pores for Cs–ItA–LCys can be described as walls rather than threads (Figure 1c). For chitosan, the polymer becomes a polyelectrolyte after the protonation of –NH_2_ groups in the presence of a weak acid. The chitosan acidic solution can be then transformed into hydrogel when it comes in contact with alkali and progressively gelifies [36]. At an acidic pH, chitosan is soluble and the subsequent adjunction of sodium hydroxide creates an instable system. The porous structure is generated during phase separation, which is induced by the brutal pH modification when immersed into NaOH and provides a less refined structure [49]. The polymer phase prevents the holes from gathering when the system is perturbed due to the pH change. Therefore, it has a structure resembling a sponge. Formed polymeric walls surrounding the solvent regions must be strong enough to prevent pores from collapsing and thick enough to maintain the porous microstructure when the solvent is removed by rinsing for neutralization [49]. Furthermore, on cryo-SEM observations of the prepared chitosan–formulation structure (Figure 2), it seems that the outer layer of the gel is compact and acts as a thick membrane. The average thickness of the outer layer is 1.20 ± 0.09 µm for the chitosan formulation (Figure 2a). The presence of this thicker membrane for CS–ItA–LCys can thus hinder aqueous permeability during immersion. A membrane was also observed for the agar gel, measuring 0.50 ± 0.24 µm (Figure 2b), which has also been observed in other published works [47].

The presence of this membrane is explained by a quick diffusion of the non-solvent (i.e., NaOH, used to rigidify the dissolved CS–ItA–LCys mixture) at the surface and then a slower diffusion of NaOH in the core of the gel [21,48]. To achieve a more homogenous layer in comparison to the inner structure, ammonium hydroxide (NH_4_OH) vapors could be used as a way to rigidify the gel while obtaining a thinner membrane, although they are more hazardous [21]. Other studies suggest that to limit this membrane formation, increasing the concentration of the basic solution used for rigidification would weaken the hydrogen bonds of the gel and allow for better exchanges with external surfaces or solutions [49]. This should be studied further in the case of the application of chitosan-based gels for the cleaning of artefacts. Indeed, this thick outer layer could inhibit the diffusion of ions within the gels and, therefore, limit the efficiency of the gel as treatment. On agar, the thin membrane is rather connected to the surface tension phenomenon, where cohesive forces between the gel polymeric chains will be stronger and denser at the edges of the gel structure to overcome the lack of surrounding similar molecules.

#### 2.1.2. Swelling Properties

**Figure 3:**
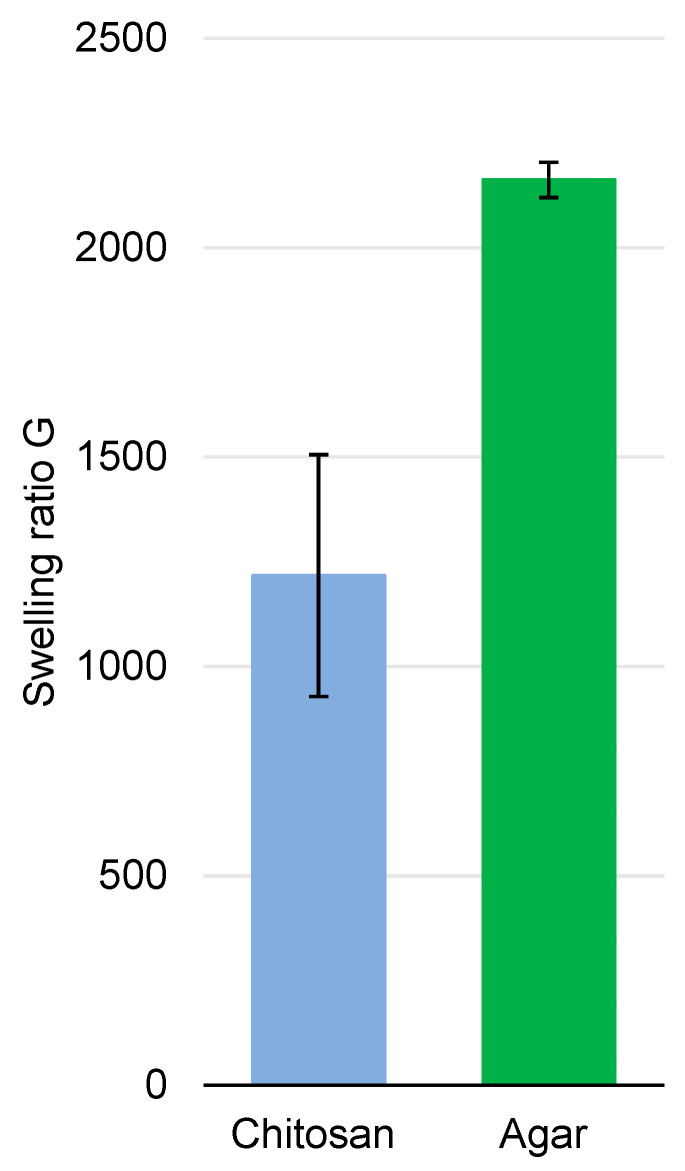
Swelling ratio of chitosan and agar gels in water.

The chitosan-based gel has a lower swelling ratio than agar gel, meaning it is able to hold in lower fractions of aqueous solutions (Figure 3). According to the literature, the swelling ratio increases with the increasing mesh size (i.e., pore diameter) [50]. When comparing two different polymers here, that is not the case.

As exposed when discussing cryo–SEM observations of the polymers, the agar network is denser, meaning there is a lower average pore diameter. However, its swelling ratio is higher (2160 ± 42 versus 1200 ± 289 for chitosan). 

#### 2.1.3. Rheological Measurements

**Figure 4:**
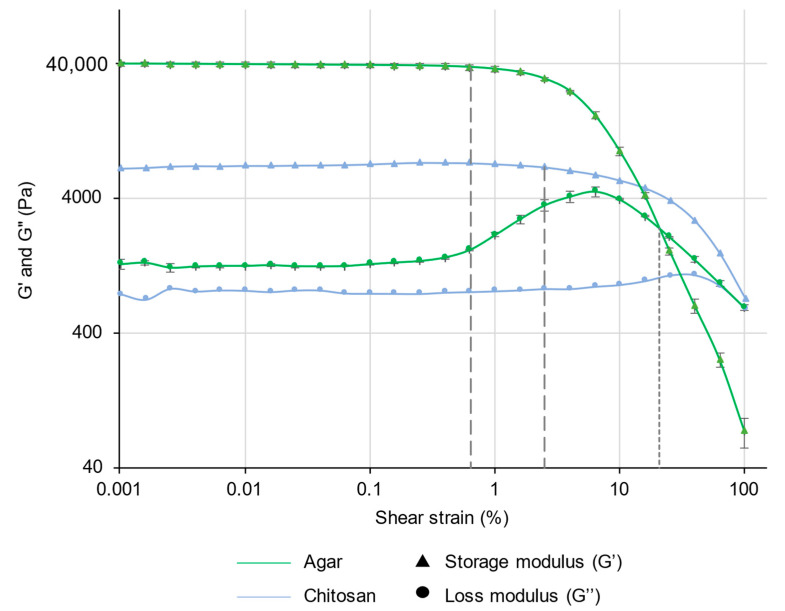
Storage (G′) and loss (G″) modulus measurements of 3% (by mass) agar gel (green) or CS–ItA–LCys gel (blue). Agar markers include error bars.

Amplitude sweep measurements of CS–ItA–LCys are compared with those of agar (Figure 4).

The linear viscoelastic (LVE) range is the interval where the storage modulus G′ and loss modulus G″ remain constant with the increasing applied deformation. This occurs in both agar and CS–ItA–LCys amplitude sweep measurements (Figure 4), demonstrating an undisturbed structure from the sample at lower deformations. The plateau of G′ describes the rigidity of the sample at rest, and the plateau of G″ is a measure of the viscosity of the gel. Here, the storage modulus G’ is higher than the loss modulus G″ for both agar and CS–ItA–LCys, indicating that the gels have a solid-like behavior [51]. Indeed, in the case of G′ > G″, the analyte behaves like a viscoelastic solid. Therefore, it can be considered to have a gel-like structure. The agar’s storage and loss modulus at the LVE range (about 44,000 and 2000 Pa, respectively) are higher than the ones of CS–ItA–LCys (about 7000 and 800 Pa, respectively), which signifies that the agar gel is more firm/rigid [51]. In addition, the greater the G′/G″ ratio between the moduli, the more the sample shows properties of a pure solid. With a G′/G″ ratio of 22 and 8.75 for agar and CS–ItA–LCys, respectively, the chitosan-based gel has a more fluid behavior than agar. This can be related to the structural aspect of chitosan. Although it possesses a thick outer membrane and walls, it displays a less dense inner network. Hence, it possesses a more flowing, less stiff behavior.

In the amplitude sweep test, there are two remarkable points:

- The yield point at the end of the LVE-region and flow point at the intersection of the curves for G′ and G″. The yield point or yield stress γ_L_ is the value of the shear stress at the limit of the LVE region. It is the moment when the applied strain starts to irreversibly damage the samples and the moduli are no longer constant.
- The flow point or flow stress, which is the value of the shear stress at the crossover point between storage and loss modulus for materials with a gel character. It is the point further in which G″ becomes greater than G′; beyond which, the material will behave as a liquid and, therefore, “flow”.

The yield stress (Figure 4 dashed lines) of agar is close to 37,000 Pa, occurring at 0.65% of shear strain, while the yield stress of CS–ItA–LCys 6700 Pa occurs at 2.5% of shear strain.

Interestingly, at the end of the LVE, the agar gel’s loss modulus G″ rises sharply at higher deformations. This phenomenon suggests an initial consistent and interconnected three-dimensional network formed by cross-linked polymers [52]. When reaching the loss modulus’s maximum, the gel breaks down starting with some microcracks. The microcrack formation results in an energy exchange that is transferred to the surrounding area as friction [52]. The rapid decrease afterwards indicates the final rupture of the gel, hence the passage to the flow area.

In reverse, the loss modulus of CS–ItA–LCys barely exhibits a bump to reach its maximum at higher shear strain values. Contrary to agar, there are no microcracks because there is no increase after the yield point.

Both gels have a yield stress occurring at close shear strains, but agar’s decrease of storage modulus G′ is more brutal, whereas the chitosan’s decrease of the storage modulus is more progressive/continuous, meaning that agar gel is more likely to break into larger pieces than CS–ItA–LCys when reaching its flow point (Figure 4, dotted line).

The chitosan-based gel’s flow point is, therefore, reached at high shear strains beyond 100%, which shows it is more resistant to deformation than agar, reaching its flow point (2394 Pa) at 20.6% of shear deformation.

Agar is more brittle than CS–ItA–LCys. This is shown by the ratio of the flow point over the yield point’s shear strains (9.3). A smaller ratio indicates a more brittle material as it means the material is destroyed as soon as it starts deforming. However, CS–ItA–LCys is more malleable (over 40).

Overall, the mechanical behavior of CS–ItA–LCys is consistent with results observed for chitosan in the literature with a progressive G’ decrease [39]. According to Montembault et al., storage modulus values in the LVE obtained for a 3% *w*/*w* chitosan gel would be circa 6000 Pa, similar to what is obtained here [53]. Similarly, the reported amplitude sweep measurement for agar provides storage modulus in a similar order of magnitude (10^4^ Pa), along with a noticeable increase of the loss modulus at the end of the LVE range [54].

Both gels, although peelable, show very different mechanical behaviors. Since CS–ItA–LCys is more flexible, it is easier to contact the surface as it is less stiff. CS–ItA–LCys’s resistance to deformation could make it fit to manipulation by conservators to apply on metal surfaces.

Studies reported the possibility of preparing a gel with agar and chitosan combined, displaying different properties than the individual pure polymers [55].

### 2.2. Amendment of Hydrogels with the Metal Uptaking Agent Deferoxamine

#### 2.2.1. Compatibility between Active Agents and Gel Preparation Protocol

**Figure 5:**
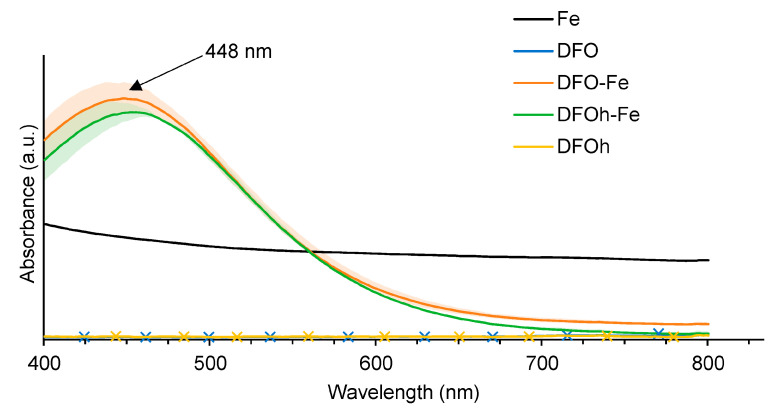
Mean UV-Visible spectrum and standard deviation (acquired in the range of 400–850 nm) of solubilized iron(III) (Fe), deferoxamine (DFO), deferoxamine after heating (DFOh), iron–deferoxamine complex (Fe–DFO) and Fe–DFO complex after the heating of deferoxamine (Fe–DFOh).

The compatibility of the chelating agent deferoxamine with gel formulations that would require a heating step, (e.g., agar) is demonstrated. The iron–deferoxamine complex is known to absorb at 448 nm [56]. From the obtained absorbance spectra (Figure 5, spectrum Fe–DFOh), it is clear that the siderophore deferoxamine still possesses chelating properties after reaching temperatures over 100 °C, confirming data from the literature [57]. Indeed, the characteristic absorbance peak of the Fe–DFO complex at 448 nm is still present.

#### 2.2.2. Cryo–SEM Imaging

**Figure 6:**
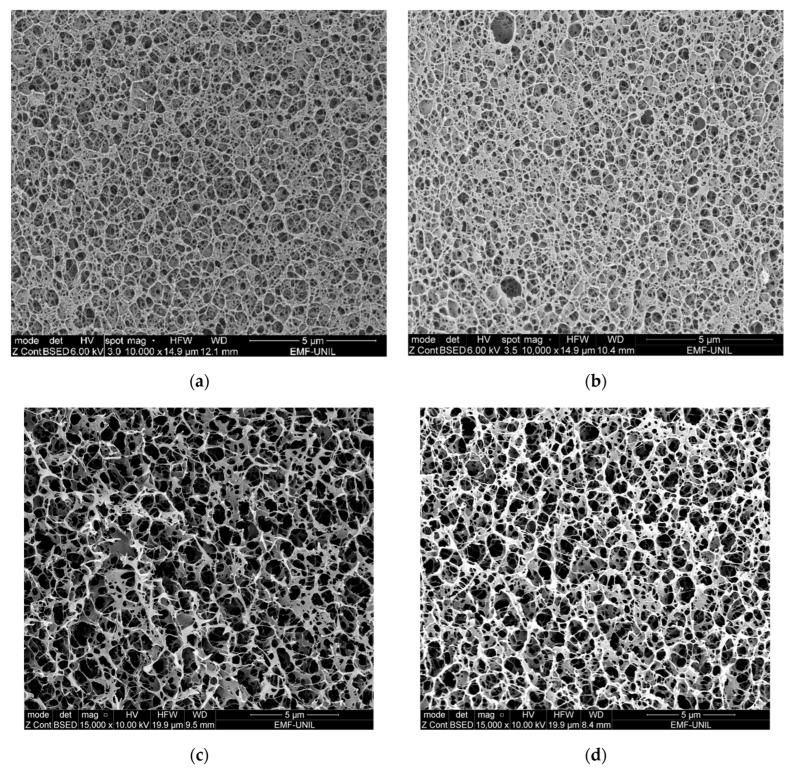
Cryo–SEM observations of (**a**) 3% (by mass) plain agar gel (**b**) 3% (by mass) agar gel amended with DFO solution, (**c**) 3.3% (by mass) plain CS–ItA–LCys (**d**) 3.3% (by mass) CS–ItA–LCys gel amended with DFO solution.

Both agar and agar–DFO gel formulations show a rather uniform network of interconnected polysaccharide chains (Figure 6a,b). The density of the network is similar, as well as the measured average pore diameter for plain agar and DFO–agar gel (0.4 ± 0.1 µm vs. 0.4 ± 0.1 µm, respectively), which means there is a similar amount of junction zones between double helices of the agar gel structure. For CS–ItA–LCys formulations (plain and DFO-amended), analogous observations are made (Figure 6c,d). However, due to the irregularity of the structure, the diameter of the pores could not be measured accurately.

Cryo–SEM observations of the matrix microstructure suggest that the metabolites do not interact with the polymers but rather stay confined in the liquid phase. The addition of siderophores does not affect the overall facies.

#### 2.2.3. Rheological Measurements

**Figure 7:**
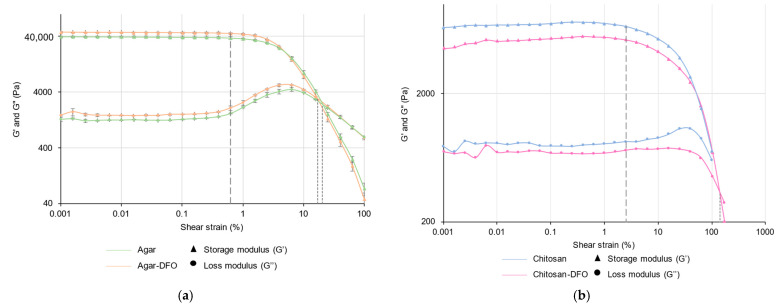
Storage (G′) and loss (G″) modulus measurements of (**a**) 3% (by mass) agar gel prepared without (orange) or with DFO amendment (green) and (**b**) 3.3% (by mass) CS–ItA–LCys gel prepared without (blue) or with DFO amendment (pink). Agar markers include error bars.

Amplitude sweep tests are plotted on Figure 7 for agar and CS–ItA–LCys formulations. For each polymer (agar and chitosan), both formulations (i.e., with or without amendment of bio-based complexants) displayed a similar linear viscoelastic range. The amplitude sweep measurements showed that the storage (G′) and loss (G″) moduli exhibit a plateau prior to the yield point, with G′ > G″. Therefore, all samples can be defined as gel-like materials.

The yield stress of agar is close to 37,000 Pa, occurring at 0.65% of shear strain, whereas values obtained for agar–DFO are about 46,000 Pa and 0.6% (Figure 7a, dashed lines).

For CS–ItA–LCys, the yield stress is close to 6700 Pa and occurs at 2.5% of shear strain, with values close to the ones obtained for CS–ItA–LCys–DFO at about 5300 Pa and 2.5% (Figure 7b, dashed lines). According to replicates, differences in values were connected to manipulation errors, namely anecdotic differences in the thickness of the gels added to minimal polymer concentration changes.

The loss modulus G″ of both formulations of agar and agar–DFO gels rise sharply at higher deformations, reaching a maximum between 4% and 6% shear strain. This means that microcracks start to develop before the complete breakdown of the sample.

Prior to a decrease, no augmentation of CS–ItA–LCys –DFO loss modulus occurs. This is contrary to the CS–ItA–LCys loss modulus, although the rise is small.

Agar–DFO possesses a flow point where the storage and loss modulus curve intercept (G′ = G″) at a shear strain of 17.1% and shear stress of 2960 Pa, which is quasi-identical to those of the agar gel (20.6%, 2394 Pa) (Figure 7a, dotted line). Regarding CS–ItA–LCys, the flow point is reached beyond 100% of shear strain in both formulations.

As inferred with electron microscopy observations, agar and CS–ItA–LCys gels would act as carriers for the metabolites’ solution.

The lack of interaction between the added molecules and the polymeric structure cannot be spread to other metal-sequestering agents. Indeed, the absence of modification of the mechanical behavior should also be considered in regard to the pH of the added solution. For instance, in the case of agar, the DFO solution has a similar pH compared to the gel, close to neutral. However, studies have proven that gel properties change slightly with pH, and that gel rigidity decreases with an important pH variation [58,59]. This was explained by the difference in the length of flexible chains from the helical network of agar gel. Gels at pH values closer to neutral have been shown to have longer and more flexible polymer chains than those with more extreme pH values. Below 5.5 and above 8, hydrolysis of the polymer reduces its molecular weight [59,60]. Consequently, the gel network’s elasticity, rigidity, and connectivity also decrease [59,61]. Short chains are stiff, correlating to the more fragile behavior of agar gel. Molecular weight reduction of agar subsequently reduces the probability to form junction zones, in addition to the flexibility of the molecular chains that would withhold the network structure of gel through interhelical association [59]. Low molecular weight chains (short chains) impede the formation of interchain bonds, i.e., the amount of hydrogen bonds formed within a junction zone would then be comparatively low [62,63]. Hence, at neutral pH, with long chains being more flexible, it can be extended further before destruction of the helical network. Therefore, it can conserve the mechanical properties of agar gels. More extreme pH values of other chelating solutions implemented in agar gels would, therefore, decrease the strain at fracture [59]. Regarding CS–ItA–LCys, it has been found that it was related to the organization of the polymer chains [64], with the increase of solution pH leading to a preference for a parallel crosslinking. Consequently, this increases the mechanical strength of the hydrogel [65].

#### 2.2.4. ATR–FTIR Spectroscopy

##### Agar

**Figure 8:**
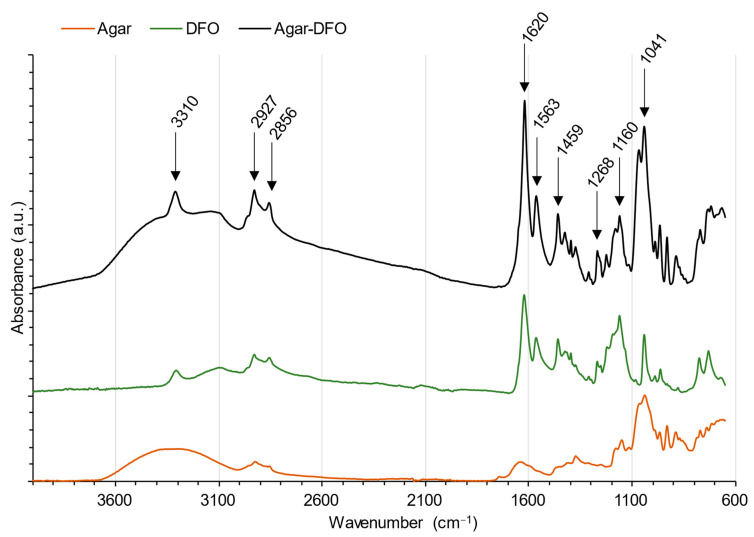
ATR–FTIR spectra of plain agar gel (orange), DFO (green) and an agar–DFO gel (black). Peaks indicated on the agar–DFO spectra are the main ones related to the presence of deferoxamine.

The obtained agar spectra displayed the typical vibrational bands of polysaccharides originated from red seaweed (Figure 8, orange spectrum). Bands at 3289 cm^−1^ and 2923 cm^−1^ are assigned to the stretching modes of OH and CH, respectively [55,66,67]. The band at 1634 cm^−1^ is attributed to the OH bending mode of water remaining in the gel, although it was dried [55,66]. The peak at 1373 cm^−1^ is assigned to ester sulfate [55]. These compounds in agar are often related to the quality of the polymer. The amount, type, and location of sulfate esters is indeed species-specific [68], but these can also be a function of other factors, including extraction methods [69]. The band at 1250 cm^−1^ stands for the S==O stretching mode [70]. The peaks observed at 1063 and 931  cm^−1^ are characteristic vibrational bands of 3,6-anhydro-galactose, corresponding to the glycosidic bond and the C–O–C bridge, respectively [55,66,67]. Bands between 800 and 900 cm^−1^ are characteristics of 3,6-anhydro-galactose network [66,70].

Regarding the deferoxamine mesylate salt spectra (Figure 8, green spectrum), the medium sharp vibrational band at 3306 cm^−1^ is attributed to the N–H bond (amine II) [71,72]. Bands at 2855 and 2928 cm^−1^ are asymmetric and symmetric stretching modes of CH_2_, respectively [71,72]. Bands at 1622, 1565, 1396, and 1268 cm^−1^ are attributed to C==O stretching from the hydroxamate (amine I), C–N stretching, N–H bending (amine II), and the O–H deformation bands, respectively [71,72,73]. Therefore, 1041, 989, and 963 cm^−1^ bands result from the stretching mode of N–O of the hydroxamate groups [71,72,73].

As can be observed on Figure 8, newly formed Agar-DFO hydrogels in the ATR–FTIR spectra are composed of a combination of peaks from the initial spectrum of DFO and respective gels, which shows no new main bonds were created, hence suggesting siderophores sit in the pores of the gels. The presence of all peaks attributed to amine, amide, or hydroxyl groups confirms the absence of new molecular bonds or functionalization through those chemical groups. Following the upload of DFO, no change could be observed on the spectra neither in terms of wavenumbers nor band shapes, strengthening the conclusion of an absence of any interaction between the gel and the chelator. Recapitulative attribution of peaks is available in Table 1. All peaks from the newly formed gels could be attributed to the initial components of the gel, including the fingerprint region (1000–600 cm^−1^) (not detailed), thus confirming that the metabolites do not interact with the polymer but rather stay confined in the liquid phase. As such, this suggests that the treatment’s reaction rate is ruled by the movement of the liquid phase inside the network and, therefore, the diffusion of DFO solution inside the gel.

##### Chitosan-Based Formulation

**Figure 9:**
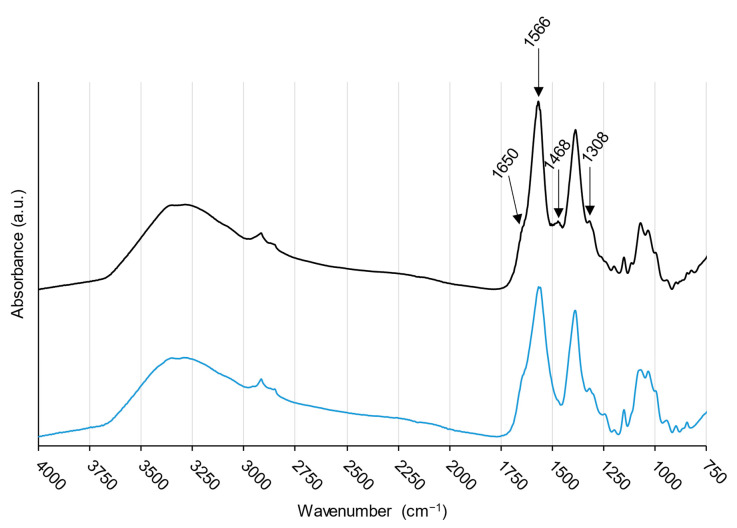
FTIR spectra of chitosan-based formulation prepared with DFO solution (black) and water (blue).

FTIR spectra of the CS–ItA–LCys formulation prepared with DFO solution (Figure 9) showed no clear peak apparition.

Indeed, most peaks from DFO overlap the ones from chitosan, in particular, the N–H and O–H in the 3000–3500 cm^−1^ region, along with peaks corresponding to primary and secondary amides (1650 and 1566 cm^−1^) and C–N stretching (1308 cm^−1^). A detailed investigation of the chitosan-based formulation FTIR spectra is proposed in Section 2.3.1.

It is worth noting that papers have loaded chitosan nanoparticles with deferoxamine for medicinal iron-uptake purposes [74]. Here, this was not reproduced as a peelable delivery system was desired. In that context, a shift in the –OH bands in FTIR spectra allowed for the conclusion that the formation of hydrogen bonds between OH or amino groups of chitosan and DFO [74]. Here, no shift was observed, with the peak for hydroxyl bond stretching at 3351 or 3293 cm^−1^ for both plain and DFO-amended chitosan. This is perhaps because the chitosan is already functionalized with the itaconic acid/L-cysteine mixture. Hence, bonds were not free to react. Interestingly, spectra collected on chitosan-based formulation amended or not with DFO showed a peak at 1468 cm^−1^ (Figure 9). This would, therefore, allow for the use of the chitosan-based formulation with the addition of secondary metabolites, which could boost the formation of chelated complexes inside the gel and, therefore, the cleaning action, in particular for iron, as more metal ions could be uptaken.

### 2.3. Inherent Capacity of CS–ItA–LCys to Uptake Silver Ions

Chitosan is a compound praised for the complexation of heavy metals and, in particular, copper ions [34,35]. Its potent iron and copper complexation has already been solidly demonstrated in the literature [38,75]. For copper, adsorption via the abundant amino and hydroxyl groups present is responsible for this ability [36,37,76,77]. 

In this paper, the chitosan-based formulation assessed was produced through a simple protocol developed by Lai et al. [39]. The formulation is composed of highly deacetylated chitosan, itaconic anhydride, and L-cysteine [39]. The last two compounds are added as they are reported to react and produce poly(thioether amide) and gelify in a straightforward way with chitosan. The presence of resulting thioether and carboxyl groups is believed to endow the hydrogel potent complexing ability to silver ions [39].

However, the method used to demonstrate silver complexation, the ICP–AES measurement of silver in the gel after immersion, is arguable as it could be due solely to the absorption of the silver ions inside the gel. Spectroscopic investigations are proposed to evaluate the molecular structure of the gel formulation and the supposedly formed silver–gel bonds.

#### 2.3.1. CS–ItA–LCys Gel Molecular Structure

**Figure 10:**
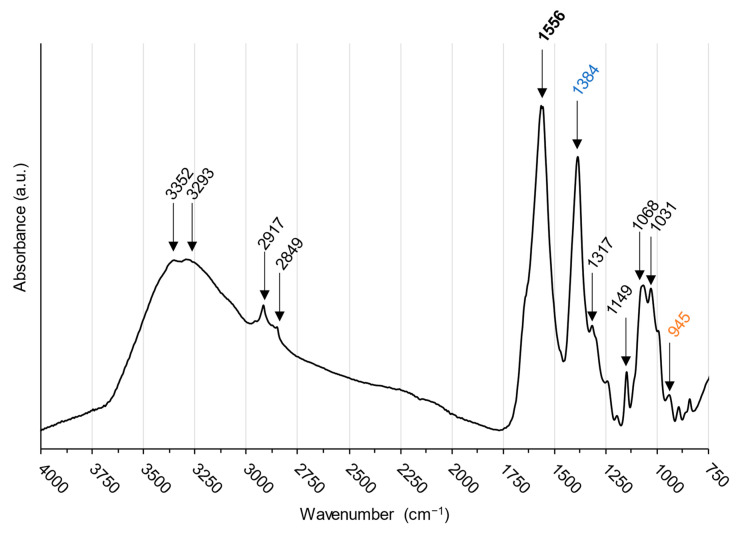
ATR–FTIR spectra of chitosan-gel prepared with itaconic anhydride and L–cysteine. Peaks in blue are related to the presence of itaconic anhydride, in orange to the presence of L-cysteine and in bold to the modification of chitosan structure.

ATR–FTIR spectroscopy was used to ascertain the molecular structure of the newly formed chitosan-based formulation, containing itaconic anhydride and L–cysteine (Figure 10).

Most peaks are assignable to chitosan’s molecule (Table 2). The broad band at 3000–3500 cm^−1^ is attributed to stretching vibrations of N–H and O–H, with small signals at 3294 and 3351 cm^−1^, respectively [78,79]. Bands at 2916 and 2848 cm^−1^ are assigned to –CH_3_ and –CH_2_ stretching vibrations, respectively [78]. Amide I and amide II bands from chitosan neckbone structure are responsible for the shoulder at 1633 cm^−1^ and an intense peak at 1556 cm^−1^, respectively, with the latter slightly shifted to lower wavenumbers from what is observed in pure chitosan [78,79,80,81]. This could be related to the addition of a new group after the amide bond formation [78,80,82]. Potentially, it could be the addition of the thioether groups mentioned previously, but so far, the nature of this new group cannot be determined. Contributions from chitosan’s C–N groups stretching vibrations are observable at 1316 cm^−1^ [81,83]. The peaks at 892, 1031, 1070, and 1150 cm^−1^ are the ones typically reported for chitosan gels’ and are related to polysaccharides [78,81,82,83,84], along with weak peaks at 1194 and 992 cm^−1^ [85].

The peak noticeable at 1384 cm^−1^ is typical of carboxyl groups present in itaconic acid structure [79].

In the literature, the presence of a signal near 1468 cm^−1^ has been linked to the thiolation of chitosan by L–Cysteine [78,80,86], supporting the fact that the functionalization of chitosan did occur as the modification of this 1468 cm^−1^ signal stands for a change in the –CH_2_ bending [87,88]. Additionally, the small peak at 1243 cm^−1^ could also be representative of thiol groups [89,90] due to the addition of L–cysteine. Furthermore, the peak at 945 cm^−1^ can be attributed to the presence of S–H in the gel, again related to the addition of L–cysteine in the formulation [24].

**Figure 11:**
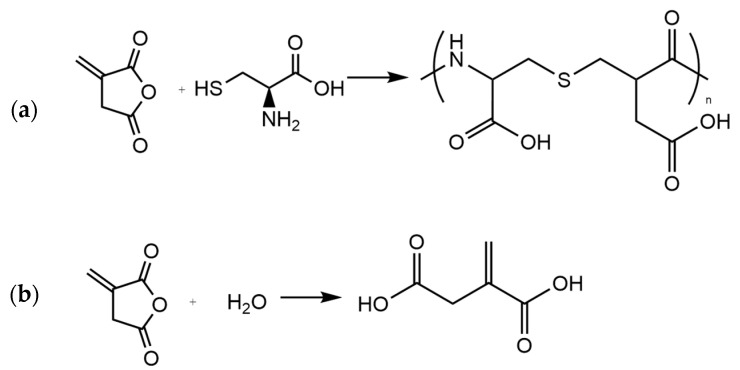
(**a**) poly(thioether amide) formation from Itaconic Anhydride and L–Cysteine, (**b**) ring opening of Itaconic anhydride into itaconic acid.

**Figure 12:**
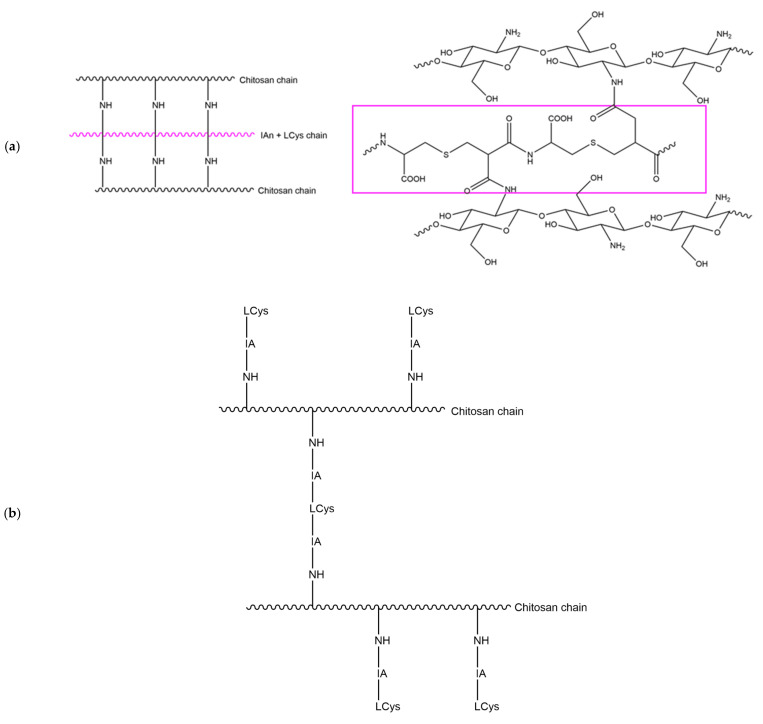
Schematic representation of chitosan functionalization through (**a**) poly(thioether amide), (**b**) successive Itaconic acid, and L–Cysteine grafting.

Although direct thiolation of chitosan has been reported in papers [80], there are more electronic displacements existing for the itaconic compounds, making them more likely to react. One possible reaction mechanism, proposed by Lai et al., is through an intermediary compound formed from L–cysteine and Itaconic anhydride (IAn). IAn and L–Cysteine would react together after IAn ring opening, which is caused by the presence of L–Cysteine. Further polymerization into poly(thioether amide) (Figure 11a) would occur, allowing this poly(thioether amide) to be grafted onto chitosan chains, linking two chitosan chains together as proposed in Figure 12a [39].

A second option could be the ring opening of IAn using the presence of water-obtaining itaconic acid (IA) (Figure 11b) [86]. IA could react with chitosan, followed by subsequent grafting of L–Cysteine to the IA side. In order to graft two chitosan chains together as proposed in the literature, there would be the need to have a molar ratio of IAn:L–Cysteine of 2:1 to allow for the subsequent grafting of another IA to previously linked L–Cysteine, which is an IA that would also attach itself to another chitosan chain (Figure 12b).

It could also be the case that most of the L–Cys/IA blocks reacted on one side mainly, and only a few of them are linking two chitosan chains together. Therefore, allowing for a near-1:1 needed molar ratio as used in the here-tested formulation, or part of the L-Cysteine could just be present floating in the gel but not necessarily attached to the rest of the structure. To obtain a more comprehensive analysis and try to understand the structure of the obtained hydrogels, other techniques might be complementary, nuclear magnetic resonance in particular [82]. The detailed investigation of the proposed mechanisms is beyond the scope of this research, but a brief depiction supporting the text is proposed in Supplementary Materials.

Spectral analyses allow for the validation of the presence of IA or L–Cysteine functional groups in the chitosan matrix. However, the complete structure cannot be confirmed based on solely FTIR spectroscopy. In any case, the presence of thiol groups and carboxyl groups in the polymer still might confer further complexing ability to the chitosan polysaccharide structure.

#### 2.3.2. Complexing Abilities

**Figure 13:**
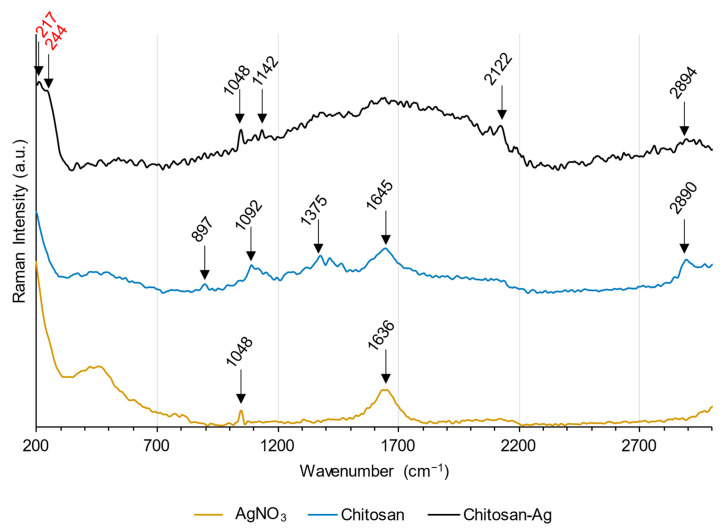
Raman spectra of AgNO_3(aq)_, Chitosan-based gel and Chitosan-based gel after immersion into AgNO_3_ solution. Peaks related to formed bonds with Ag are in red.

The complexation capacity of the CS–ItA–LCys gel with silver is assessed by Raman spectroscopy. Most bands from the Raman spectra of CS–ItA–LCys immersed into the AgNO_3_ solution can be attributed to silver nitrate or plain chitosan (Figure 13); in particular, the band at 1048 cm^−1^ is typical of silver nitrate [87]. The band at 1641 cm^−1^ is believed to be a combination of the bands at 1636 cm^−1^ and 1645 cm^−1^ ascribed to silver nitrate’s NO^3−^ and chitosan’s N–C=O, respectively [88,89,90]. The band at 2890 cm^−1^ is interpreted as stretching vibrations of CH_2_ groups from chitosan [88,91]. 

On the bare chitosan-based gel’s spectrum, bands at 897, 1092, and 1375 cm^−1^ are typical Raman shifts for chitosan [88,91].

**Figure 14:**
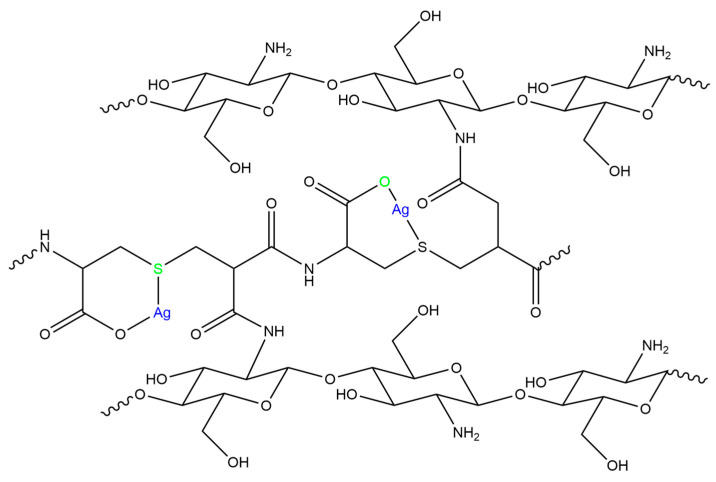
Chitosan chelation of silver ions as proposed in literature [39].

Two remarkable new signals were observed: a small peak at 217 cm^−1^, representative of the vibration mode of the Ag–O [92], and a shoulder at 244 cm^−1^, which is ascribed to the vibration mode of Ag–S bonds [93]. Therefore, FT–Raman analysis confirmed the existence of new bonds involving both silver and atoms from CS–ItA–LCys, thus proving the complexing potential of the chitosan formulation tested and coming as a support for the molecular structure of CS–ItA–LCys chelating silver proposed in the literature (Figure 14).

As a perspective, similar characterization was attempted to test agar–silver molecular interactions and should be continued.

Previous work on interactions between hydrogel networks and targeted metal ions are mostly based on comparative quantitative studies, assessing the concentration of metal uptaken into the gel matrix with the use of Inductively Coupled Plasma (ICP) or Atomic Absorption Spectroscopy (AAS), but not addressing the possible mechanisms of the interaction [39,41]. Notably, one paper discussed the interaction between the agar structure and copper using Electron paramagnetic resonance (EPR) spectroscopy [19]. Here, the interaction between CS–ItA–LCys and Ag at the molecular level is demonstrated using FT–Raman spectroscopy.

The obtained results were evaluated while considering the molecular structure of the chitosan formulation, in particular, the presence of binding groups. This was done previously by Guaragnone et al., who reported the presence of carboxylates in the studied polymer (PAA) [41]. For such, other polymers might be of particular interest in the perspective of gel networks displaying inherent complexing abilities, for example, gellan gum, which also has carboylic acid moieties.

Chitosan confirmed antimicrobial, protective, and chelating properties that make it a promising candidate for the application on composite objects composed of metal and organic materials where biodeterioration is a concern. In addition, these chelating abilities are of interest for the removal of metal stains from organic substrates (i.e., paper, fabrics). 

Although further research is necessary to evaluate the effect of the complexing property on near-insoluble silver corrosion products (Ag2S), applications could be foreseen on brocades, for instance, to remove copper or silver tarnish from the metallic threads. Additionally, it would be of interest to verify the actual need of L–Cysteine and itaconic anhydride adjunction in the chitosan formulation to obtain the silver complexing ability.

## 3. Conclusions

The objectives of this study were to assess biobased gels, either as chelator’s carriers or complexing gels for metal uptake treatments.

Comparing agar and CS–ItA–LCys, structural differences were observed between agar and via cryo–SEM, showing a more regular structure for agar and the presence of a relatively thick membrane on the CS–ItA–LCys outer layer. Mechanical distinctions could also be highlighted using amplitude sweep, showing a stiffer and more brittle behavior for set agar gel. Further work might be conducted, studying other natural polymers (e.g., sodium alginate, gellan gum) or a combination of polymers, such as, for example, agar–chitosan gel.

Optical microscopy, molecular spectroscopy, and rheology data showed the compatibility of both agar and CS–ItA–LCys with deferoxamine, with the absence of modifications upon addition of the natural chelator. Compatibility with other chelators should also be investigated independently of the here-obtained results.

The tangible proof of created Ag–O and Ag–S bonds over the complexation of silver ions by CS–ItA–LCys was obtained by FT–Raman spectroscopy. This finding is relevant in the context of cultural heritage conservations treatments, in particular, metal uptake.

This is a first step into the formulation of gels with inherent complexing abilities, therefore avoiding the use of additional chemicals or agents, as well as simplifying the preparation step by removing some of the components.

These findings must be pushed forward for a potential application in cultural heritage on actual insoluble corrosion products encountered on objects, e.g., silver sulfides. 

The outcomes of such studies could be of interest to other fields of application for wastewater treatment and bioremediation of metal contaminated sites.

## 4. Materials and Methods

### 4.1. Gels Preparation

#### 4.1.1. Agar

**Figure 15:**
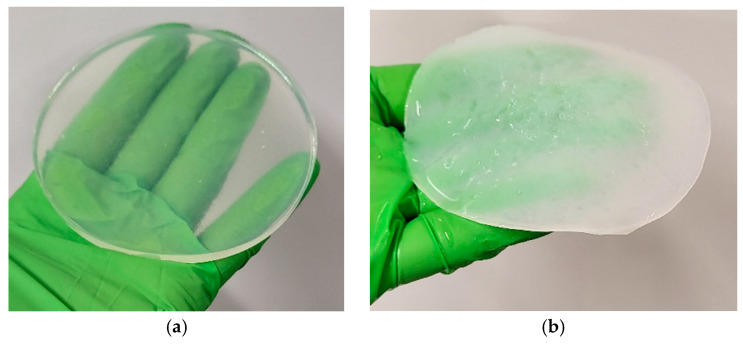
(**a**) 3% *w*/*v* agar gel after preparation (**b**) 3.3% *w*/*v* chitosan-based gel after preparation.

Three-percent (by mass) agar gels were prepared in either milli–Q water or a 3 × 10^−2^ M deferoxamine (DFO) (Desferal^®^, Novartis, Basel, Switzerland) solution. The mixtures were boiled, cooled down twice, and then molded carefully to obtain a 3 mm thick gel. The double-heating technique has been shown to improve the mechanical properties [6]. Obtained gel can be observed on Figure 15a. 

#### 4.1.2. Chitosan-Based Gels

Ninety-five-percent deacetylated chitosan (Chitoscience Chitosan 95/200 from Heppe Medical Chitosan GmbH, Saale, Germany), itaconic anhydride, and L–cysteine (Merck, Germany) were ground in proportion 1:1:1 in a mortar. The formulation is composed of highly deacetylated chitosan, Itaconic anhydride, and L–Cysteine [39]. The last two compounds are added as they are reported to react and produce poly(thioether amide) and gelify in a straightforward way with chitosan [39].

The mixture was mixed with milli–Q water or a 3 × 10^−2^ M DFO solution in a 10% (by mass) proportion, hence leading to a 3.3% (by mass) of chitosan in the final gel. This allows for the obtainment of a viscous gel, with chitosan solubilizing and gelling under the action of the weak itaconic acid formed by reaction of itaconic anhydride with water [79,86]. The viscous gel was centrifuged at 5000 rpm for 5 min to remove air bubbles and then poured slowly/carefully into a mold to obtain a 3 mm thick gel. NaOH 3 M was poured in the mold onto the gel and left for 2 h in order to obtain a rigid gel. The gel was then thoroughly rinsed with milliQ water to neutralize [21]. More precisely, the gel was immersed into milliQ water, which changed twice per day for 5 days. 

Obtained gel can be observed on Figure 15b.

### 4.2. Gels Characterization for POLYMER Comparison and/or Active Agents Addition Evaluation

#### 4.2.1. Cryo–SEM Imaging

SEM imaging was performed in order to observe the supramolecular structure of the gels. Samples of agar and chitosan gels were prepared following the procedure, which allows for the conservation of the microstructure of the matrix, as described by Rabhani et al. [94]. Cryo–SEM experiments were performed using a PP3010 cryo-transfer system (Quorum, Laughton, UK) using a Quanta FEG250 SEM (FEI, Hillsboro, OR, USA). Small samples (10 × 5 mm) of agar or chitosan gels were placed on an aluminum stub using carbon conductive glue, and the stub was secured on the specimen holder. The sample was then rapidly immersed into liquid nitrogen and transferred into the preparation chamber under a vacuum. A fractured surface of the gel was obtained by hitting the top part of the sample with a knife inside the chamber. The sample was then sublimed inside the SEM chamber, allowing for water removal without distortions and potential subsequent artifacts during imaging. To avoid charging problems, the sample was sputter-coated with platinum. In all cases, the imaging was performed using an accelerated voltage of 6–10 kV and a working distance of 7.9–13.6 mm. Image analysis was performed using ImageJ 1.53o software.

#### 4.2.2. Swelling Properties

The swelling ratio provides the ratio between the mass of the final hydrogel and the mass of the two components in the initial mixture, which can be calculated using the equation:

(1) $$G=\frac{Ws-Wd}{Wd}\times 100$$

where *Wd* is the weight of the dry hydrogel and *Ws* is the weight of the swollen gel after preparation [95]. Gels were weighted immediately after preparation and placed in an oven at 70 °C for 8 h, then they were weighted. The measurements were performed on six replicates.

#### 4.2.3. Rheological Measurements

To investigate the mechanical properties of hydrogel formulations, rheology was performed, in particular, by using the amplitude sweep technique. This technique evaluates viscoelastic systems (e.g., pastes, gels). The amplitude of the oscillatory shear strain (i.e., deformation) is gradually increased at a constant frequency. The resulting stress (storage (G′) and loss (G″) moduli) is plotted as a function of the shear strain. G′ and G″ provide information about the behavior of the studied system according to its elastic and viscous fractions, respectively [51]. Amplitude sweep was assessed using a MCR 302e rheometer (Anton Paar, Buchs, Switzerland) with a 25 mm profiled plate–plate measuring system, thus avoiding the slipping effect, at a temperature set at 25 °C. A normal force of 1 N and frequency of 10 Hz were applied to the analyzed gel samples. Two measurements were performed on each gel sample. Displayed data for agar are the mean of the obtained results and for chitosan are the most representative of the two measurements.

Further investigation could be conducted to confirm this result, in particular, frequency sweeps to verify G′ > G″.

#### 4.2.4. Fourier Transformed Infrared Spectroscopy

Spectra (4000–650 cm^−1^) of dried gels were acquired. Gels were dried in an oven for 24 h at 35 °C. An iS5 Thermo Scientific spectrometer (ThermoFisher, Strasbourg, France) was used with a diamond-attenuated total reflectance (ATR) crystal plate (iD5 ATR accessory), collecting 16 scans at a resolution of 4 cm^−1^. Collection and data processing were conducted with Omnic 9.2.86 software. Baseline and atmospheric corrections were performed on the resulting spectra to remove residual signatures of atmospheric CO_2_ and H_2_O.

### 4.3. Compatibility between Active Agents and Gel Preparation Protocol

Compatibility of added agents with heat-requiring protocols for gels preparation was evaluated using an Ultra–Violet–Visible (UV–Vis) spectrophotometer VICTOR Nivo Multimode Microplate Reader from Perkin Elmer (Bülach, Switzerland). In particular, the heating resistance of the siderophore, deferoxamine (DFO) (Desferal^®^, Novartis, Basel Switzerland), was examined. For such, equimolar (10^−2^ M) solutions (100 µL) of ferric nitrate (Fe(NO_3_)_3_·9H_2_O) and DFO were mixed, both before and after boiling of the siderophore solution. UV–Visible spectra of the mixture, diluted if necessary, were acquired in the visible range 400–850 nm. 

### 4.4. FT–Raman Spectroscopy for Gel-Complexing Abilities

This technique is the coupling of a Raman accessory with an FTIR instrument. Raman measurements were performed with a emission wavelength of 1064 nm, allowing for a reduction of the fluorescence effect and a more direct correlation between the observed vibrational bands and molecular bonds [96].

Here, it was used to assess the complexing abilities of the chitosan gel regarding silver ions and, therefore, evaluate the bonds forming between the chitosan gel and silver ions. Prepared chitosan gel samples were immersed in 20 mM AgNO_3_ solution for 24 h and then stocked in milli–Q water. Chelation abilities of the gel after immersion were analyzed using an FT–Raman (Bruker RFS100 with a continuous YAG laser at 1064 nm as the source). Gel samples, swollen, were analyzed with a laser power of 500 mW in the range of 50–4000 cm^−1^ and with between 100 and 300 accumulations to obtain a signal-to-noise ratio sufficient to ascertain peak detection. No particular sample preparation was performed for FT–Raman analysis.

## Figures and Tables

**Table 1:** Assigned ATR–FTIR bands for Agar gel, DFO, and Agar–DFO gel. Relative band height is indicated by the first letter w = weak, m = medium, s = strong, v = very. Shape is indicated by sharp, br = broad, or shoulder.

Band Assignment	Wavenumber (cm^−1^)		
	Agar	Deferoxamine	Agar-DFO
N–H stretch.		3306 m, sharp	3310 m, sharp
–OH	3289, br		3137, br
C–N–H		3099 w, br	
CH_2_ as. Stretch.		2928 vw, sharp	2927 w, sharp
–CH	2923 w, sharp		
CH_2_ s. stretch.		2855 w, sharp	2856 vw, sharp
C==O stretch.		1622 vs, sharp (hydroxamate)	1620 vs, sharp
O–H bend.	1634 m		
C–N–H		1565 m, sharp	1563 m, sharp
CH_3_		1459 m, sharp	1459 m, sharp
C–H		1425 w	1426 w
O–H deform.		1396 w, sharp	1396 w, sharp
–C–N stretch.	1314 vw		1310 vw
C–N stretch N–H bend.		1268 vw	1268 vw1254, shoulder
S==O	1252 vw		
C–N stretch.		1161 m	1160 m
N–O stretch.		1041 vs, sharp 989 w, sharp 963 m, sharp	1041 vs 989 w, sharp 966 m, sharp
C==O (3,6-anhydro-a-L-galactose)	931 m, sharp		931 m, sharp

**Table 2:** Assigned absorption bands of one-pot chitosan–itaconic acid L–cysteine formulation. Relative band height is indicated by the first letter w = weak, m = medium, s = strong, v = very. Shape is indicated by sharp, br = broad or shoulder. With one asterisk are the peaks related to the presence of itaconic acid and two asterisks related to the presence of L-cysteine. In bold are the peaks suggesting modification of chitosan’s structure.

Band Assignment	Wavenumber (cm^−1^)
O–H stretching	3351 m, br
N–H stretching	3293 w, br
CH_3_ stretching	2917 s, sharp
CH_2_ stretching	2849 w, sharp
C=O amide I	1633 w, shoulder
**–NH_2_ amide II**	**1556 vs, sharp**
**–CH_2_ bending**	**1468 vw, shoulder**
C=O carboxylate group *	1384 vs, sharp
C–N stretching	1317 w, br
C–SH **	1243 w
C–O–C	1149 m, sharp
C–O stretching	1068 m, sharp
C–O stretching	1031 m, sharp
S–H	945 vw, sharp
C–H bending	895 w shoulder

## Data Availability

The data presented in this study are available on request from the corresponding author. A specific link is necessary to access data from the repository.

## Supplementary Materials

The following supporting information can be downloaded at: https://www.mdpi.com/article/10.3390/gels10010055/s1, Figure S1: Possible reaction of chitosan with itaconic anhydride and L-cysteine.
