# Supplementary material for: Agar and Chitosan Hydrogels’ Design for Metal-Uptaking Treatments
Source: Gels. 2024 Jan 11;10(1):55. doi: 10.3390/gels10010055 (PMC10815442; doi:10.3390/gels10010055)
Supplement: Supplementary file 1 [file gels-10-00055-s001.zip › gels-2732025-supplementary.pdf]

Supplementary Materials

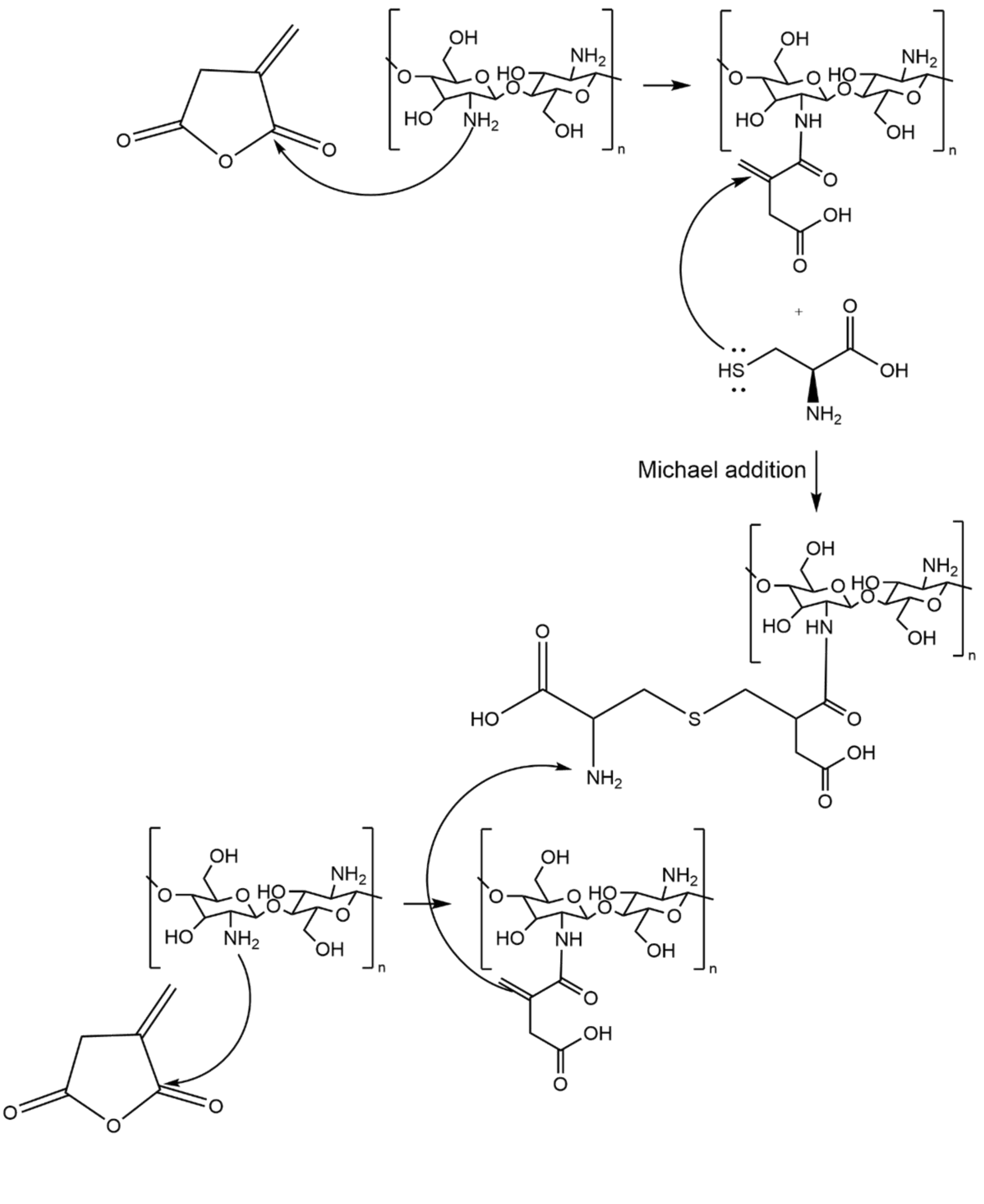

Figure S1: Possible reaction of chitosan with itaconic anhydride and L-cysteine.
